# Endodontic microbiology

**DOI:** 10.4103/0972-0707.73386

**Published:** 2010

**Authors:** L Lakshmi Narayanan, C Vaishnavi

**Affiliations:** Department of Conservative Dentistry & Endodontics, SRM Kattankulathur Dental College & Hospital, SRM University, Chennai, India

**Keywords:** Bacterial infections, biofilms, dental pulp microbiology, *Enterococcus faecalis*, gram positive bacteria, periapical periodontitis/microbiology

## Abstract

Root canal therapy has been practiced ever since 1928 and the success rate has tremendously increased over the years owing to various advancements in the field. One main reason is the complete understanding of the microbiology involved in the endodontic pathology. This has helped us to modify the conventional treatment plans and effectively combat the microorganisms. Now, studies are aiming to explore the characteristics of the “most” resistant organism and the methods to eliminate them. This article gives an insight of the microbiology involved in endodontic pathology and discusses its role in our treatment procedure. Information from original reviews listed in PubMed, published from 1995 to 2010, has been mainly included in this review.

## INTRODUCTION

Preservation of teeth by endodontic therapy has gained lot of popularity because of increased and predictable success rate of our endodontic procedures, the reason for this being the complete understanding of endodontic pathology and our ability to combat the same. Essentially, endodontic infection is the infection of the dental root canal system and the major etiologic agent of apical periodontitis.[1] Although various chemical and physical factors can induce periradicular inflammation, scientific evidence clearly indicates that microorganisms are essential for the progression and perpetuation of different forms of apical periodontitis.[2]

The rationale for endodontic treatment is to eradicate the infection, to prevent microorganisms from infecting or re-infecting the root and/or periradicular tissues. Thus, a thorough understanding of the endodontic microbiota associated with different forms of disease is the basis for the success of endodontic treatment. This article, based on the search made in PubMed, briefs about the endodontic microbiology and its importance for the success of endodontic treatment.

## PATHWAYS OF INFECTION

It is proved beyond doubt that presence of microbiota is a major deterrent in endodontic infection by the classical study by Kakehashi *et al*.[2] There are so many ways by which the microorganisms reach the pulp and it is of prime importance that we know the same for our treatment planning. The various routes by which the microorganisms reach the pulp are as follows.[3]

Dentinal tubules: After a carious lesion or during dental procedures, microorganisms may use the pathway in a centripetal direction to reach the pulp. Bacteria gain access to the pulp when the dentin distance between the border of carious lesion and the pulp is 0.2 mm.[4]

Open cavity: Direct pulp exposure of traumatic origin such as in coronal fracture, or that of iatrogenic nature due to operative procedures, breaks the physical barrier imposed by dental structures and leaves pulp in contact with the septic oral environment.

Periodontal membrane: Microorganisms from gingival sulcus may reach the pulp chamber through the periodontal membrane, using a lateral channel or the apical foramen as a pathway. This pathway becomes available to microorganisms during a dental prophylaxis, due to dental luxation, and more significantly, as a result of the migration of epithelial insertion to the establishment of periodontal pockets.

Blood stream: A transient bacteremia may occur for any number of reasons during the normal day of a healthy individual. The bacteria present in the blood would be attracted to the dental pulp following trauma or operative procedure that produced inflammation without causing pulp exposure. This attraction through blood or lymph is known as anachoresis, which serves as a path for endodontic infection.

Faulty restoration: Studies have proven that salivary contamination from the occlusal aspect can reach the periapical area in less than 6 weeks in canals obturated with guttapercha and sealer.[5] If the temporary seal is broken or if the tooth structure fractures before final restoration, or if the final restoration is inadequate, bacteria may gain access to the periapical tissue and result in infection.

Extent: Microorganisms might reach the principal and/or lateral canals migrating from an infected tooth to a healthy pulp as a consequence of the contiguousness of the tissues, thereby spreading the infection to an adjacent tooth.

## CORRELATION OF MICROBES TO INFECTION

Almost 700 bacterial species can be found in the oral cavity, with any particular individual harboring 100–200 of these species.[6] Once the root canal is infected coronally, infection progresses apically until bacterial products or bacteria themselves are in a position to stimulate the periapical tissues, thereby leading to apical periodontitis. Endodontic infections have a polymicrobial nature, with obligate anaerobic bacteria conspicuously dominating the microbiota in primary infections. There are various microorganisms related to intra-radicular and extraradicular infections and organisms involved in persistent infection. They are discussed below.

### Intraradicular infections

The endodontic pathogens that cause the primary intraradicular infections are the following.

1) Black pigmented Gram negative anaerobic rods include species formerly known as *Bacteroides melaninogenicus*. These bacteria have been reclassified into two genera: (a) saccharolytic species – *Prevotella* and (b) asaccharolytic species – *Porphyromonas*.[7]

*Prevotella* species detected in endodontic infections include

- Prevotella intermedia
- Prevotella nigrescens
- Prevotella tannerae
- Prevotella multissacharivorax
- *Prevotella baroniae* and
- Prevotella denticola.

*Porphyromonas* species detected in endodontic infections include

- *Porphyromonas endodontalis* and
- Porphyromonas gingivalis.

2) *Tannerella forsythia* (previously called *Bacteroides forsythus* or *Tannerella forsythenis*) was the first periodontal pathogen to be detected in endodontic infection.[8]

3) *Dialister* species are asaccharolytic obligately anaerobic Gram negative coccobacilli which have been consistently detected in endodontic infections.

- *Dialister pneumosintes* and
- Dialister invisus.

4) *Fusobacterium* is also a common member of endodontic microbiota.

- Fusobacterium nucleatum
- Fusobacterium periodonticum

5) Spirochetes are highly motile, spiral-shaped, Gram negative bacteria with periplasmic flagella. All oral spirochetes fall into the genus *Treponema*.[9] Prevalent species are

- Treponema denticola
- Treponema sacranskii
- Treponema parvum
- Treponema maltophilum and
- Treponema lecithinolyticum.

6) Gram positive anaerobic rods have also been found in endodontic microbiota like

- Pseudoramibacter alactolyticus
- Filifactor alocis
- *Actinomyces* spp.
- Propionibacterium propionicum
- *Olsenella* spp.
- Slackia exigua
- *Mogibacterium timidum* and
- *Eubacterium* spp.

7) Gram positive cocci that are present in endodontic infection:

- *Parvimonas micra* (previously called *Peptostreptococcus micros* or *Micromonas micros*)
- *Streptococcus* spp. which include,
  Streptococcus anginosus
  Streptococcus mitisi
  Streptococcus sanguinis
- *Enterococcus faecalis*.

Other bacterial spp. which are present in low to moderate values include

- *Campylobacter* spp. which are Gram negative anaerobic rods; common species are,
  *Campylobacter rectus* and
  *Campylobacter gracilis*.
- *Catonella morbic* which is a saccharolytic obligate anaerobic Gram negative rod
- Veillonella parvula
- Eikenella corrodens
- Granulicatella adiacens
- Neisseria mucosa
- Centipeda periodontii
- Gemella morbillorum
- Capnocytophaga gingivalis
- Corynebacterium matruchotii
- *Bifidobacterium dentium* and
- anaerobic lactobacilli.

Apart from these, several uncultivated phylotypes which can be unrecognized but play a role in pathogenesis of apical periodontitis, such as[10]

- *Dialister* oral clone BSO16
- *Migasphaera* oral clone BSO16
- Solobacterium
- Olsenella
- Eubacterium
- Cytophaga
- Lachnospiraceae oral clone 55A-34
- *Veillonella* oral clone BP 1–85
- Bacteroidetes oral clone XO 83
- *Prevotella* oral clone PUS 9.180
- *Eubacterium* oral clone BP 1–89 and
- Lachnospiraceae oral clone MCE 7–60.
- Other microorganisms in endodontic infections

Fungi – particularly, *Candida* spp. (e.g.,) *Candida albicans*

Archaea – These are diverse group of prokaryotes which are distinct from bacteria. They are traditionally recognized as extremophiles but recently these microorganisms are found to thrive in non-extreme environment including human body. Methanogenic archaea have been detected in periodontal disease and chronic apical periodontitis.[11]

Viruses – Viruses are particles structurally composed of a nucleic acid molecule (DNA or RNA) and a protein coat. These viruses require viable host cells to infect and use the cell’s machinery to replicate the viral genome. Hence, they cannot survive in a necrotic root canal.

The presence of viruses in the root canal has been reported only for non-inflamed vital pulps of patients infected with human immunodeficiency virus and herpes viruses where living cells are found in abundance.[12,13] Among the *Herpes* spp., the human cytomegalovirus and Epstein–Barr virus may be implicated in the pathogenesis of apical periodontitis.

### Extraradicular infections

Intraradicular microorganisms usually constrain themselves in the root canal due to the defense barrier. In specific circumstances, microorganisms can overcome this defense barrier and establish an extraradicular infection. This may lead to development of acute apical abscess with purulent inflammation in periapical tissue. The extraradicular infections are dependent on or independent of an intraradicular infection. The dominant microorganisms present are anaerobic bacteria[14–17] like

- *Actinomyces* spp.
- Propionibacterium propionicum
- *Treponema* spp.
- Porphyromonas endodontalis
- Porphyromonas gingivalis
- Treponema forsythia
- *Prevotella* spp. and
- *Fusobacterium nucleatum*.

### Bacteria persisting intracanal disinfection procedures and after root canal treatment

Some microorganisms are resistant to antimicrobial treatment and can survive in the root canal after biomechanical preparation.

The most common Gram negative anaerobic rods are

- Fusobacterium nucleatum
- *Prevotella* spp. and
- *Campylobacter rectus*.

The most common Gram positive bacteria are

- Streptococci (*Streptococcus mitis, Streptococcus gordonii, Streptococcus anginosus, Streptococcus oralis*)
- Lactobacilli (*Lactobacillus paracasei* and *Lactobacillus acidophilus*)
- Staphylococci
- E. faecalis
- Olsenella uli
- Parvimonas micra
- Pseudoramibacter alactolyticus
- *Propionibacterium* spp.
- *Actinomyces* spp.
- *Bifidobacterium* spp. and
- *Eubacterium* spp.

Sometimes, yeasts, commonly *C. albicans*, are also found in small amounts.

*E. faecalis* and yeast, mainly *C. albicans*, has been repeatedly identified as the species most commonly recovered from root canals undergoing retreatment, in cases of failed endodontic therapy and canals with persistent infections.[18,19] *E. faecalis* are gram positive cocci and facultative anaerobes. They are normal intestinal organisms and may inhabit the oral cavity and gingival sulcus. When this bacterium is present in small numbers, it is easily eliminated; but if it is in large numbers, it is difficult to eradicate. *E. faecalis* has many distinct features which make it an exceptional survivor in the root canal. These microorganisms can perform the following.

- Live and persist in poor nutrient environment
- Survive in the presence of several medications (e.g., calcium hydroxide) and irrigants (e.g., sodium hypochlorite)
- Form biofilms in medicated canals
- Invade and metabolize fluids within the dentinal tubules and adhere to collagen
- Convert into a viable but non-cultivable state
- Acquire antibiotic resistance
- Survive in extreme environments with low pH, high salinity and high temperatures
- Endure prolonged periods of starvation and utilize tissue fluid that flows from the periodontal ligament

## PATHOPHYSIOLOGY

The human commensal microbiota populates the mucosal surface of the oral cavity, gastrointestinal tract, urogenital tract and surface of the skin. This commensal microbiota, which has coevolved with its host, has acquired the means of surviving and tolerating host defense mechanisms.[20,21] However, when the host is compromised, or if invading microorganisms are sufficiently pathogenic, disease can develop. Pathogenicity refers to the ability of an organism to cause disease in another organism. These organisms are known as pathogens which include bacteria, fungi, viruses, protozoa and parasites. These pathogens are capable of adhering, colonizing, surviving, propagating, at the same time evading host defense mechanisms such as neutrophils, complement and antibodies. In addition, they can cause tissue destruction directly or indirectly.[22] Direct tissue damage can be induced by enzymes, exotoxins and metabolites. Indirect tissue damage can be induced from a host immune reaction capable of causing tissue destruction that is stimulated by bacterial components which include lipopolysaccharide (LPS), peptidoglycan (PG), lipoteichoic acid (LTA), fimbriae, outer membrane proteins, capsular components and extracellular vesicles. The degree of pathogenicity or disease producing ability of a microorganism is known as virulence. Several physicochemical factors in the root canal have the potential to influence the pathogenicity of bacteria, which include the degree of anaerobiosis, pH level, the availability of exogenous and endogenous nutrients, as well as the surfaces available for adherence like dentin. In infected root-filled teeth, any medicament remnants and root filling material are additional factors to influence pathogenicity.

### Virulence factors

Many microorganisms found in endodontic infections are commensals in the oral cavity, which have gained entry into the pulp tissue of the root canal typically via the caries process. Identification and characteristics of specific virulence factors that might play a role in endodontic infections are discussed here.

LPS: This is also known as endotoxin.[23] LPS is an integral part of cell wall of Gram negative bacteria. When released, LPS has numerous biologic effects including the mobilization of inmunosurveillance mechanisms in the pulp. These endotoxins are associated with pulpal pain, periapical inflammation, activation of complement and periapical bone destruction.[24–27]

PG: PG is the major component of Gram positive cell wall. Upon cell lysis, PG is released and can react with the innate immune system as well as induce upregulation of proinflammatory and anti-inflammatory cytokines in T cells.[28] PG may facilitate an adaptive immune response via macrophages.[29] The potency of PG is strongly boosted in the presence of LPS.[30]

LTA: LTA is a cell wall component of Gram positive bacteria, composed of echoic acid and lipid.[31] LTA shares many of its pathogenic properties with LPS.[32] LTA is released as a result of cell lysis and binds to target cells, which then interacts with circulating antibodies and activates complement cascade and cause damage.

Fimbriae: Fimbriae are long, filamentous macromolecules found on the surface of many Gram negative bacteria. The thin hair-like projections are made of protein subunits (they are distinct from flagella). Fimbriae are involved in attachment to surfaces and interactions with other bacteria.[33]

Capsules: A capsule is a well-organized layer outside the cell wall of the bacteria, generally composed of polysaccharides and other materials. Capsules serve to facilitate protection of the bacterial cell against desiccation, phagocytosis, bacterial viruses and hydrophobic toxic materials such as detergents. Bacteria and fungi utilize capsule formation to inhibit complement activation and resist ingestion by phagocytes.

Extracellular vesicles: Extracellular vesicles are produced by Gram negative bacteria and allow the release of their products into the extracellular environment. The contents include proteins and lipids that are involved in a diverse array of activities including hemagglutination, hemolysis, bacterial adhesion and proteolytic activities.[34] Extracellular vesicles are a means by which bacteria interact with prokaryotic and eukaryotic cells and can modulate interactions between neighboring bacteria.[35,36]

Exotoxins: Exotoxins are toxins released by a living cell, which can trigger excessive and aberrant activation of T cells.[37] Bacterial toxins can also target other microorganisms, e.g., bacteriocins, proteinaceous toxins produced by bacteria are bacteriostatic or bacteriocidal to other bacteria.[38]

Extracellular proteins: Many of these extracellular proteins are enzymes which are produced by bacteria. These enzymes are released during bacterial cell lysis which contributes to spread of infection, including proteases that neutralize immunoglobulins and complement components.[39] Enzymes like hyaluronate lyase, chondroitin sulphatase, beta glucuronidase, DNase and acid phosphatase contribute to tissue disintegration.

Short-chain fatty acids: These are major by-products of fermentation process performed by obligate anaerobes, and include butyric acid and propionic acid. These acids stimulate the inflammatory response and inflammatory cytokine release which contribute to infection process.[40,41]

Polyamines: Polyamines are small, polycationic molecules like putrescine, cadaverine, spermidine and spermine which contribute to clinical symptoms like pain (including percussion pain) and formation of sinus tract.[42] These polyamines act by modulating a variety of ion channels.[43]

Superoxide anions: Superoxide anions are biologically toxic and highly reactive free radicals. These are produced by few bacterial species and also by the cells of immune system. They cause lysis of erythrocytes[44] and are involved in interspecies interaction.

However, diverse arrays of virulence factors are available to modulate the participation of microorganisms in host–microbe interactions. An absolute cause and relationship occurs between the virulence factors and clinical signs and symptoms in root canal infections. Apart from these, there are additional mechanisms by which the microorganisms might modulate the infection process, which include the ability of some intracellular bacteria to inactivate the killing mechanisms of phagocytic cells and thereby avoid being killed by macrophages and neutrophils.[45] In addition, some bacteria can genetically vary their surface antigens, thus causing difficulty for the immune system to target these organisms.[46] A thorough understanding of these virulence factors helps to identify the therapeutic targets in endodontic infections.

## IMPORTANCE OF UNDERSTANDING MICROBIOLOGY FOR THE SUCCESS OF NON-SURGICAL AND SURGICAL ENDODONTIC TREATMENT

The presence of microorganisms in the dental pulp is directly associated with the development of periapical disease. Chemomechanical preparation of the infected root canal using antimicrobial agents, followed by obturation and coronal restoration, provides a favorable outcome. However, failure of root canal treatment sometimes occurs due to persistent or secondary intraradicular infection.[47,48]

Microorganisms found in failed endodontically treated teeth have either remained in the root canal from previous treatment or have entered during or after treatment via leakage. It is difficult to differentiate between the microorganisms remaining from primary infections and new microorganisms contributing to the secondary infection. The remaining microorganisms from primary infection should have maintained the viability throughout the treatment procedure. This might occur as a result of an inability of chemomechanical instrumentation and because of inaccessible locations of bacteria in isthmuses, accessory canal and apical regions of canals.[49]

Success of non-surgical endodontic treatment is limited by the heterogeneity of patients and difficulty in maintaining standardized clinical conditions. Thus, a thorough knowledge and understanding of these persistent endodontic microbes helps us to decide on surgical treatment or retreatment.

## BACTERIAL BIOFILMS

Biofilm is a mode of microbial growth where dynamic communities of interacting sessile cells are irreversibly attached to a solid substratum, as well as to each other, and are embedded in a self-made matrix of extracellular polymeric substances.[50] The microorganisms living in a community must have the following four basic criteria:[51]

1. possess the abilities to self-organize (autopoiesis),
2. resist environmental perturbations (homeostasis),
3. be more effective in association than in isolation (synergy) and
4. respond to environmental changes as a unit rather than single individuals (communality).

### Development of biofilm

Bacteria can form biofilms on any surface that is bathed in a nutrient-containing fluid. The three major components involved in biofilm formation are bacterial cells, a solid surface and a fluid medium.

Biofilm formation occurs in three stages given below.

Stage 1: Adsorption of inorganic and organic molecules to the solid surface occurs, leading to the formation of conditioning layer. th

Stage 2: Adhesion of microbial cells to the conditioned layer: There are many factors that affect the bacterial attachment like pH, temperature, surface energy of the substrate, nutritional availability, time of contact of bacteria, bacterial cell surface charge and surface hydrophobicity. The bacteria substrate interaction occurs in three phases:

- Phase 1: Transport of microbe to substrate surface which is mediated by fimbriae, pili, flagella and extracellular polysaccharides (glycocalyx).
- Phase 2: Initial non-specific microbial–substrate adherence which occurs due to combination of electrostatic attraction, covalent and hydrogen bonding, dipole and hydrophobic interaction.
- Phase 3: Specific microbial substrate adherence phase. In this phase, adhesin or ligand on the bacterial cell surface binds to receptors on the substrate.

Stage 3: Development of biofilm and biofilm expansion occurs. In this stage, monolayer of microbes attracts secondary colonizers forming microcolony, and the collection of microcolonies gives rise to the final structure of biofilm.[52,53]

### Endodontic biofilms

Endodontic microbiota is established to be less diverse compared to oral microbiota. Progression of infection alters the nutritional and environmental status within the root canal, making it more anaerobic with depleted nutritional levels. These changes offer a tough ecological niche for the surviving microorganisms. But complete disinfection of root canal is very difficult to achieve because of persistent microbes in anatomical complexities and apical portion of root canal. Because biofilm is the manner of bacterial growth which survives unfavorable environmental and nutritional conditions, the root canal environment will favor biofilm formation.

Endodontic bacterial biofilms can be categorized as

- intracanal biofilms,
- extraradicular biofilms,
- periapical biofilms and
- biomaterial-centered infections.

#### Intracanal microbial biofilms

They are microbial biofilms formed on the root canal dentin of an endodontically infected tooth.[54]

#### Extraradicular microbial biofilms

They are also termed as root surface biofilms which are formed on the root (cementum) surface adjacent to the root apex of endodontically infected teeth.[46]

Extraradicular biofilms are reported with asymptomatic periapical periodontitis and in chronic apical abscesses with sinus tracts. Sometimes, the extraradicular biofilm becomes calcified and gets associated with periapical inflammation and delayed periapical healing in spite of adequate orthograde root canal treatment.[55]

#### Periapical microbial biofilms

They are isolated biofilms found in the periapical region of endodontically infected teeth. Periapical biofilms may or may not be dependent on the root canal. These microorganisms have the ability to overcome host defense mechanisms, thrive in the inflamed periapical tissue and subsequently induce a periapical infection.[56]

#### Biomaterial-centered infection

Biomaterial centered infection is caused when bacteria adhere to an artificial biomaterial surface and form biofilm structures.[57] Presence of biomaterials in close proximity to the host immune system can increase the susceptibility to biofilm. In endodontics, biomaterial-centered biofilms form on root canal obturating materials. These biofilms can be intraradicular or extraradicular depending on whether the obturating material is within the root canal or has extruded beyond the root apex.

## CONCLUSION

Infection of the root canal is not a random event. The type and mix of the microbial flora develop in response to the surrounding environment. Microorganisms that establish in the untreated root canal experience an environment of nutritional diversity. In contrast, well-filled root canal offers the microbial flora a small, dry, nutritionally limited space. Thus, we should obtain a better understanding of the characteristics and properties of bacteria and their biofilms along with the environmental changes, to enhance success.

## References

[1] Siqueira JF, PittFord T (2008). Microbiology of apical periodontitis. Essential endodontology.

[2] Kakehashi S, Stanley HR, Fitzgerald RJ (1965). The effects of surgical exposures of dental pulps in germ-free and conventional laboratory rats. Oral Surg Oral Med Oral Pathol.

[3] Bammann LL, Estrela C (2009). Microbiological aspects in endodontics: Endodontic Science.

[4] Dahlen G, Moller A, Slots J, Taubman MA (1991). Microbiology of endodontic infection. Contemporary Oral Microbiology and immunology.

[5] Torabinejad M, Ung B, Kettering J (1990). Invitro bacterial penetration of coronally unsealed endodontically treated teeth. J Endod.

[6] Paster BJ, Olsen I, Aas JA, Dewhirst FE (2000). The breadth of bacterial diversity in the human periodontal pocket and other oral sites. Periodontol.

[7] Shah HN, Collins DM (1990). Prevotella, a new genus to include *Bacteroides melaninogenicus* and related species formerly classified in the genus Bacteroides. Int J Syst Bacteriol.

[8] Conrads G, Gharbia SE, Gulabivala K, Lampert F, Shah HN (1997). The use of a 16S rDNA directed PCR for the detection of endodontopathogenic bacteria. J Endod.

[9] Dahle UR, Titterud Sunde P, Tronstad L (2003). Treponemas and endodontic infections. Endod Top.

[10] Sakamoto M, Rocas IN, Siqueira JF, Benno Y (2006). Molecular analysis of bacteria in asymptomatic and symptomatic endodontic infections. Oral Microbiol Immunol.

[11] Vianna ME, Conrads G, Gomes BP, Horz HP (2006). Identification and quantification of archaea involved in primary endodontic infections. J Clin Microbiol.

[12] Glick M, Trope M, Bagasra O, Pliskin ME (1991). Human immunodeficiency virus infection of fibroblasts of dental pulp in seropositive patients. Oral Surg Oral Med Oral Pathol Oral Radiol Endod.

[13] Slots J (2000). Herpes viruses in periodontal diseases. Periodontol.

[14] Sunde PT, Olsen I, Debelian GJ, Tronstad L (2002). Microbiota of periapical lesions refractory to endodontic therapy. J Endod.

[15] Sunde PT, Tronstad L, Eribe ER, Lind PO, Olsen I (2000). Assessment of periradicular microbiota by DNA – DNA hybridization. Endod Dent Traumatol.

[16] Tronstad L, Barnett F, Riso K, Slots J (1987). Extraradicular endodontic infections. Endod Dent Traumatol.

[17] Gatti JJ, Dobeck JM, Smith C, White RR, Socransky SS, Skobe Z (2000). Bacteria of asymptomatic periradicular endodontic lesions identified by DNA – DNA hybridization. Endod Dent Traumatol.

[18] Love RM (2001). Enterococcus faecalis - a mechanism of its role in endodontic failure. Int Endod J.

[19] Gopikrishna AV, Kandaswamy D, Jeyavel RK (2006). Comparative evaluation of the antimicrobial efficacy of five endodontic root canal sealers against *Enterococcus faecalis* and *Candida albicans*. J Conserv Dent.

[20] Henderson B, Wilson M (1998). Commensal communism and the oral cavity. J Dent Res.

[21] Moine P, Abraham E (2004). Immunomodulation and sepsis: Impact of the pathogen. Shock.

[22] Lawrence JG (2005). Common themes in the genome strategies of pathogens. Curr Opin Genet Dev.

[23] Schein B, Schilder H (1975). Endotoxin content in endodontically involved teeth. J Endod.

[24] Horiba N, Maekawa Y, Yamauchi Y, Ito M, Matsumoto T, Nakamura H (1992). Complement activation by lipopolysaccharides purified from root canals. Oral Surg Oral Med Oral Pathol.

[25] Dwyer TG, Torabinejad M (1980). Radiographic and histologic evaluation of the effect of endotoxin on periapical tissues of the Cat. J Endod.

[26] Khabbaz MG, Anastasiadis PL, Sykaras SN (2001). Determination of endotoxins in the vital pulp of human carious teeth: Association with pulpal pain. Oral Surg Oral Med Oral Pathol Oral Radiol Endod.

[27] Jacinto RC, Gomes BP, Shah HN, Ferraz CC, Zaia AA, Souza-Filho FJ (2005). Quantification of endotoxins in necrotic root canals from symptomatic and asymptomatic teeth. J Med Microbiol.

[28] Wang JE, Jørgensen PF, Almlöf M, Thiemermann C, Foster SJ, Aasen AO (2000). Peptidoglycan and lipoteichoic acid from Staphylococcus aureus induce tumor necrosis factor alpha, interleukin 6 (IL-6) and IL-10 production in both T cells and monocytes in a human whole blood model. Infect Immun.

[29] Myhre AE, Aasen AO, Thiemermann C, Wang JE (2006). Peptidoglycan - a endotoxin in its own right?. Shock.

[30] Wang JE, Jørgensen PF, Ellingsen EA, Almiöf M, Thiemermann C, Foster SJ (2001). Peptidoglycan primes for LPS – induced release of proinflammatory cytokines in whole human blood. Shock.

[31] Hogg SD, Whiley RA, De Soet JJ (1997). Occurrence of Lipoteichoic acid in oral streptococci. Int J Syst Bacteriol.

[32] Cohen J (2001). Mechanisms of tissue injury in sepsis: Contrasts between gram positive and gram negative infection. J Chemother.

[33] Tang G, Yip HK, Samaranayake LP, Chan KY, Luo G, Fang HH (2004). Direct detection of cell surface interactive forces of sessile, fimbriated and non-fimbriated Actinomyces spp. using atomic force microscopy. Arch Oral Biol.

[34] Kinder SA, Holt SC (1989). Characterization of coaggregation between *Bacteroides gingivalis* T22 and *Fusobacterium nucleatum* T18. Infect Immun.

[35] Beveridge TJ (1999). Structures of gram-negative cell walls and their derived membrane vesicles. J Bacteriol.

[36] Kuehn MJ, Kesty NC (2005). Bacterial outer membrane vesicles and the host-pathogen interaction. Genes Dev.

[37] Llewelyn M, Cohen J (2002). Superantigens: Microbial agents that corrupt immunity. Lancet Infect Dis.

[38] Tomita H, Fujimoto S, Tanimoto K, Ike Y (1997). Cloning and genetic and sequence analyses of the bacteriocin 21 determinant encoded on the *Enterococcus faecalis* pheromone – responsive conjugative plasmid pPDI. J Bacteriol.

[39] Sundqvist G, Carlsson J, Herrmann B, Tarnvik A (1985). Degradation of human immunoglobulins G and M and complement factors C3 and C5 by black-pigmented bacteroides. J Med Microbiol.

[40] Niederman R, Zhang J, Kashket S (1997). Short chain carboxylic acid Stimulated, PMN – mediated gingival inflammation. Crit Rev Oral Biol Med.

[41] Kurita-Ochiai T, Hashizume T, Yonezawa H, Ochiai K, Yamamoto M (2006). Characterization of the effects of butyric acid on cell proliferation, cell cycle distribution and apoptosis. FEMS Immunol Med Microbiol.

[42] Maita E, Horiuchi H (1990). Polyamine analysis of infected root canal contents related to clinical symptoms. Endod Dent Traumatol.

[43] Thomas T, Thomas TJ (2001). Polyamines in Cell growth and cell death: Molecular mechanisms and therapeutic applications. Cell Mol Life Sci.

[44] Falcioni GC, Coderoni S, Tedeschi GG, Brunori M, Rotilio G (1981). Red cell lysis induced by microorganisms as a case of superoxide and hydrogen peroxide dependent hemolysis mediated by oxyhemoglobin. Biochim Biophys Acta.

[45] Jansen A, Yu J (2006). Differential gene expression of pathogens inside infected hosts. Curr Opin Microbiol.

[46] Frank SA, Barbour AG (2006). Within-host dynamics of antigenic variation. Infect Genet Evol.

[47] Nair PN (2006). On the causes of persistent apical periodontitis, a review. Int Endod J.

[48] Siqueira JF (2001). Aetiology of root canal treatment failure: Why well-treated teeth can fail. Int Endod J.

[49] Nair PN, Henry S, Cano V, Vera J (2005). Microbial status of apical root canal system of human mandibular first molars with primary apical periodontitis after “one-visit” endodontic treatment. Oral Surg Oral Med Oral Pathol Oral Radiol Endod.

[50] Costerton JW, Lewandowski Z, DeBeer D, Caldwell D, Korber D, James G (1994). Biofilms, the customized microniche. J Bacteriol.

[51] Caldwell DE, Atuku E, Wilkie DC, Wivcharuk KP, Karthikeyan S, Korber DR (1997). Germ theory vs. community theory in understanding and controlling the proliferation of biofilms. Adv Dent Res.

[52] Costerton J, Stewart PS, Greenberg EP (1999). Bacterial biofilm: A common cause of persistent infections. Science.

[53] Cowan M, Taylor KG, Doyle RJ (1987). Energetics of the initial phase of adhesion of streptococcus sanguis to hydroxyapatite. J Bacteriol.

[54] Nair P (2000). Apical periodontitis: A dynamic encounter between root canal infection and host response. Periodontol.

[55] Harn WM, Chen YH, Yuan K, Chung CH, Huang PH (1998). Calculus-like deposit at apex of tooth with refractory apical periodontitis. Endod Dent Traumatol.

[56] Hornef M, Wick MJ, Rhen M, Normark S (2002). Bacterial strategies for overcoming host innate and adaptive immune responses. Nat Immunol.

[57] Wilson M (1996). Susceptibility of oral bacterial biofilm to antimicrobial agents. J Med Microbiol.

